# Early warning signals of infectious disease transitions: a review

**DOI:** 10.1098/rsif.2021.0555

**Published:** 2021-09-29

**Authors:** Emma Southall, Tobias S. Brett, Michael J. Tildesley, Louise Dyson

**Affiliations:** ^1^ The Zeeman Institute for Systems Biology and Infectious Disease Epidemiology Research, School of Life Sciences and Mathematics Institute, University of Warwick, Coventry CV4 7AL, UK; ^2^ Mathematics for Real World Systems Centre for Doctoral Training, Mathematics Institute, University of Warwick, Coventry CV4 7AL, UK; ^3^ Odum School of Ecology, University of Georgia, Athens, GA, USA; ^4^ Center for the Ecology of Infectious Diseases, University of Georgia, Athens, GA, USA

**Keywords:** critical transitions, disease emergence, disease elimination, early warning signals, time-series signals, critical slowing down

## Abstract

Early warning signals (EWSs) are a group of statistical time-series signals which could be used to anticipate a critical transition before it is reached. EWSs are model-independent methods that have grown in popularity to support evidence of disease emergence and disease elimination. Theoretical work has demonstrated their capability of detecting disease transitions in simple epidemic models, where elimination is reached through vaccination, to more complex vector transmission, age-structured and metapopulation models. However, the exact time evolution of EWSs depends on the transition; here we review the literature to provide guidance on what trends to expect and when. Recent advances include methods which detect when an EWS becomes significant; the earlier an upcoming disease transition is detected, the more valuable an EWS will be in practice. We suggest that future work should firstly validate detection methods with synthetic and historical datasets, before addressing their performance with real-time data which is accruing. A major challenge to overcome for the use of EWSs with disease transitions is to maintain the accuracy of EWSs in data-poor settings. We demonstrate how EWSs behave on reported cases for pertussis in the USA, to highlight some limitations when detecting disease transitions with real-world data.

## 1. Introduction

Infectious diseases contribute to nearly one-third of the worldwide disease burden [1]. Advances in public health over the twentieth century have resulted in major successes, including the elimination of smallpox, and over a 99% reduction in the incidence of poliomyelitis since 1988 [2]. However, the continuous threat posed by newly emerging diseases strains public health resources and widens inequality gaps within countries. Infectious diseases disproportionately affect individuals from low-income countries and can trigger social and economical instability [3]. In many cases, infectious diseases are treatable with existing medicines or are preventable with vaccines, yet they continue to persist, causing significant harm and death. Understanding when a disease has been eliminated is a topic of global health and economic importance. If successful, limited resources for disease management, such as vaccines, can be reallocated, and the usage of highly toxic treatments can end. However, if infectious disease control is eased prematurely it could reverse all progress towards disease elimination and result in disease resurgence. Early detection of emergent events can allow for effective disease management and potential containment, limiting the total burden of disease.

Epidemiologists are interested in identifying two critical transitions. Firstly, a key problem in infectious disease management is assessing if a disease has been eliminated, thus prompting the end of a control campaign. Secondly, the ability to assess the potential epidemic threat posed by emerging and re-emerging diseases. The basic reproduction number, *R*_0_, is an important epidemiological quantity for assessing the threat of a disease; when *R*_0_ < 1 the disease-free state is stable and disease is unable to sustain itself without repeated extrinsic introductions, while *R*_0_ > 1 indicates that sustained transmission (including epidemics and endemicity) is possible. Epidemiologists seek to identify when a disease is approaching the critical threshold at *R*_0_ = 1. Traditional mathematical modelling of infectious diseases allows researchers to analyse the local stability properties and capture the system’s sensitivity to changes. However, there are many known, and sometimes fundamental, limitations of mathematical modelling, including the following.

— Model sensitivity: Does the model accurately represent the problem being addressed? What are the model assumptions? Could this model be surjective, i.e. could the same dynamics be observed by two completely different models?
— Parameter sensitivity: How will the model be fitted to the data? Can all unknown parameters in the model be fitted? How does the model handle missing data?
— Computational cost: Can results be produced quickly, in real time, as more data become available?
— Generality to different environments, locations and problems.

Model-independent methods, which aim to avoid these issues, do not rely on empirically fitted models and have grown in popularity to support evidence of disease emergence and disease elimination. A variety of statistical methodologies have been proposed for detecting anomalies in data in the context of detecting infectious disease outbreaks [4,5]. These surveillance-based approaches identify patterns of disease outbreaks as they arise in public health data to inform the implementation of control, specifically methods which provide sufficient time to allow interventions to take place.

Early warning signals (EWSs) are a proposed model-independent method for detecting critical transitions, rooted in the mathematical theory of dynamical systems, and their use has been increasing throughout the twenty-first century. EWSs are a group of statistical time-series signals that change in a consistent way on the approach to a bifurcation, which can be detected in time-series and spatial data. Methodological approaches that detect the critical transition *R*_0_ = 1 in time-series data can provide early evidence of infectious disease outbreaks, and also can inform the path to disease elimination. EWSs offer a potential computationally inexpensive and efficient method for monitoring the status of a disease, by detecting when a disease system shifts abruptly from one stable state to the other. Nearly all previous work in traditional statistical surveillance has focused on detecting ongoing disease outbreaks [6], while the generality of detecting critical transitions with EWSs offers a dual purpose: to detect disease emergent events and disease elimination.

Critical transitions are a feature of many complex systems. The potential for using EWSs to anticipate events before they occur is invaluable, and offers the ability for researchers to change the future course of a system. Research in the dynamical properties of a system on the approach to a critical transition has a long history, from the first proposal of monitoring the return time of a system to signal a tipping point [7] to Hohenberg & Halperin's [8] review of critical phenomena known as ‘critical slowing down’ (CSD). More recently, the phenomenon has been identified as increased autocorrelation, variance and magnitude of fluctuations as a system approaches a transition, owing to the system’s slow recovery from perturbations as its dominant eigenvalue approaches zero. Since real-world systems are subject to noise, this phenomenon can be detected indirectly from an increasing ‘memory’ in stochastic fluctuations, resulting in changes in statistical indicators (or EWSs) such as variance and autocorrelation [9]. Much of the previous literature on EWSs has focused on ecological [10–13] and climate systems [14–16], exploring leading indicators of ecosystem collapse and sudden climatic shifts, respectively; reviewed in [9]. While these indicators have successfully predicted transitions in data [15,17] and model simulations [18,19], potential indicators typically perform well for some systems and poorly for others [20].

The specific characteristics of epidemiological transitions (e.g. that they are associated with transcritical bifurcations) and data (e.g. aggregated case reports subject to under-reporting) have therefore required multiple theoretical studies extending the literature [21–31]. Table 1 gives an overview of the literature in EWSs of disease transitions, summarizing all published papers on this topic by transition studied (elimination or emergence), with a focus on the type of infectious disease time-series data used (incidence, prevalence or other) and whether the observed indicators exhibited a rising or falling trend prior to the critical transition. Bold typeface highlights if an observed indicator was shown to be reliable prior to an epidemiological transition. While some studies found EWSs, there are disagreements among papers of similar epidemiological systems.

**Table 1.** A summary of the EWS literature with epidemiological applications (up to 2021).

title (*first author*)	date	ref.	data	data type	CT	*V*/s.d.	*M*	CV	AC	ID	other
Theory of early warning signals of disease emergence and leading indicators of elimination (*S. O’Regan*)	Aug 2013	[21]	Sim.	Prev.	Em and Ext	+/ −		**+**	**+**		significant only for Ext
Leading indicators of mosquito-borne disease elimination (*S. O’Regan*)	Sep 2016	[22]	Sim.	Prev.	Ext	**+**=/**−**		**+**	+		
Monitoring the path to the elimination of infectious diseases (*J. Drake*)	June 2017	[32]	Sim.	Inc	Ext			**+**			
Anticipating the emergence of infectious diseases (*T. Brett*)	July 2017	[23]	Sim.	Prev.	Em	**+**	**+**	=	**+**	**+**	correlation time (**+**) and Shannon entropy (**+**)
Forecasting infectious disease emergence subject to seasonal forcing (*P. Miller*)	Sep 2017	[33]	Sim.	Inc.	Em	**∗**	**∗**	*	*	*	variance convexity (**∗**) and autocovariance (**∗**)
Critical dynamics in population vaccinating behaviour (*D. Pananos*)	Dec 2017	[34]	Real	*	Em	**+**		**+**	+		*Twitter and Google trends
How stochasticity influences leading indicators of critical transitions (*S. O’Regan*)	Jun 2018	[24]	Sim.	Prev.	Ext	+/ −		+/ −	+		
Anticipating epidemic transitions with imperfect data (*T. Brett*)	Jun 2018	[35]	Sim.	Inc and Prev.	Em	**+**	**+**	=	**+**	**+**	
Detecting early-warning signals of influenza outbreak based on dynamic network marker (*P. Chen*)	Jul 2018	[36]	Real	Inc.	Em	**+**					dynamic network marker increases (**+**)
Spatial correlation as an early warning signal of regime shifts in a multiplex disease–behaviour network (*P. Jentsch*)	Jul 2018	[37]	Sim.	Prev. *	Em						* and % of vaccinated individuals. Lag-1 spatial correlation (**+**)
Disentangling reporting and disease transmission (*E. O’Dea*)	Mar 2019	[25]	Sim.	Inc	Em	+	+	−	+		decay rate (**−**)
The statistics of epidemic transitions (*J. Drake*)	May 2019	[26]	Sim.	Prev.	Em	−			+		variance of susceptibles (+)
The problem of detrending when analysing potential indicators of disease elimination (*A. Dessavre*)	Nov 2019	[27]	Sim.	Prev.	Ext	**+**		**+**			
Early warning signals of malaria resurgence in Kericho, Kenya (*M. Harris*)	Mar 2020	[38]	Real	Inc.	Em	**+**	+	+	**+**	+	decay time (**+**) and first difference (**+**/**−**)
Detecting critical slowing down in high-dimensional epidemiological systems (*T. Brett*)	Mar 2020	[28]	Sim.	Inc.	Em	**+**	**+**	+/ −	+	+	
Dynamical footprints enable detection of disease emergence (*T. Brett*)	Mar 2020	[39]	Sim and Real	Inc.	Em						composite EWS (emergence risk **+**)
Spatial early warning signals of social and epidemiological tipping points in a coupled behaviour–disease network (*B. Phillips*)	May 2020	[29]	Sim	Prev.	Em						dissimilar joint count (**+**), mutual information (**−**), Moran’s *I* (**+**), Geary’s *C* (+/ =)
Transient indicators of tipping points in infectious diseases (*S. O’Regan*)	Sep 2020	[30]	Sim	Inc. and Prev	Em and Ext						reactivity (+) and amplification envelope (+)
Anticipating the novel coronavirus disease (COVID-19) pandemic (*T. Kaur*)	Sep 2020	[40]	Real	Inc.	Em	**+**			+		
Prospects for detecting early-warning signals in discrete event sequence data: application to epidemiological incidence data (*E. Southall*)	Sep 2020	[31]	Sim.	Inc. and Prev.	Em and Ext	**+**/**−**		**+**/**−**	+		KT (**−**) for prev. (Ext CT)
Predicting local COVID-19 outbreaks and infectious disease epidemics based on landscape network entropy (*R. Liu*)	Mar 2021	[41]	Real	Inc.	Em						landscape network entropy (**+**)
Performance of early-warning signals for disease emergence: a case study on COVID-19 data (*D. Proverbio*)	Pre-print	[42]	Real	Prev	Em	+			+		
Early warning signals predict emergence of COVID-19 waves (*D. O’Brien*)	Pre-print	[43]	Real	Inc.	Em	**+**			**+**		composite EWS

Note: This table shows whether the data used in each publication were real or simulated (Sim); for prevalence (prev.) or incidence (inc.) and whether they consider the critical transition (CT) of emergence/re-emergence (Em) or elimination (Ext). In this table, the common EWSs compared are variance and standard deviation (*V*/s.d.); mean (*M*); coefficient of variation (CV); autocorrelation lag-1 (AC), index of dispersion (ID) and first difference of variance (FD). Other EWSs such as recovery rate, skewness, decay time, autocovariance and kurtosis are included in the 'other' column if the paper found them indicative. A ‘+’ in the table indicates the EWS was found to be increasing, whereas a ‘−’ indicates a decreasing trend. A ‘+/ −’ indicates that the EWS is increasing/decreasing under some circumstances (e.g. only reliable for a certain data type or only for one critical transition). Bold text indicates significant or robust indicators. Constant trend is given by a ‘=’, if a trend is increasing (or decreasing) then constant then ‘+=’ (‘−=’) is given. A ‘*’ is given when the trend of the EWS is not described, but its reliability or performance is discussed.

Initial research was restricted to theoretical exploration, determining signals that are exhibited prior to a transition in synthetic epidemiological data [21–31]. There are many barriers to the development of EWSs with empirical epidemiological data. Recently, research attention has moved towards incorporating more realistic synthetic data, including reporting errors, and the implementation of EWSs on incidence-type data [31,33,35,44]. The first validation analysis on empirical datasets was conducted in [39] and supports the potential of EWSs with real-world data. This article will summarize the findings on the suitability of EWSs being used in epidemiology, and in particular will discuss future avenues such as the application of these methods to benefit programme managers for disease control.

### 1.1. Terminology

*Bifurcation*. The term which describes a shift in the qualitative behaviour of a dynamical system’s steady state, caused by changes in parameter values. The presence of a bifurcation point can result in small parametric changes leading to abrupt system state changes.

*Critical slowing down* (*CSD*). The phenomenon that a system closer to a critical transition will take more time to recover from perturbations, owing to the dominant eigenvalue approaching zero.

*Early warning signals* (*EWSs*). Measurable manifestations of CSD in dynamical time series. Candidate EWSs are useful if they change in a consistent way on the approach to a critical transition, such as rising autocorrelation and variance in the fluctuations about a system’s steady state (also known as statistical indicator or leading indicator).

*Time of detection and lead time.* The first time that the statistical signature of CSD is detected, and if the time of the ‘true’ critical transition is known, the lead time is the lag between the time of detection and reaching the bifurcation.

*Sensitivity and specificity of EWSs.* Performance of indicators, determined by their ability to detect a critical transition in time series going through a transition (true-positive rate; sensitivity), and their ability to *not* detect a transition in time series that is not undergoing a transition, e.g. null datasets (true-negative rate; specificity).

## 2. Analytical derivations of EWSs

The effects of CSD can be appreciated mathematically by deriving the statistical indicators analytically. O’Regan & Drake [21] first presented a derivation of EWSs for epidemiological systems, such as the classic one-dimensional SIS model (susceptible–infected–susceptible model; see [45]). The statistical indicators of the SIS system were first analysed at steady state, and then were extended for the continuous system [27]. The system models the prevalence of an infectious disease over time, which can be described by the deterministic equation

2.1 $$\begin{array}{rl} \frac{dI}{dt} & =\beta (1-I)I-\gamma I\\ & =\beta I\left(1-\frac{1}{R_{0}}-I\right), \end{array}$$

where *β* is the transmission rate of the disease and *I* is the prevalence at time *t*. This system has two fixed points: the disease-free state (I* = 0), which is stable when *R*_0_ < 1, and the endemic steady state (I* = 1 − (1/*R*_0_)), which is stable when *R*_0_ > 1. This system can be transformed into the transcritical bifurcation normal form by a change of variables that rescale time (e.g. *t*′ = *βt*), such that

$$\frac{dx}{dt}=x(r_{t}-x),$$

where *r*_*t*_ = 1 − (1/*R*_0_) is the bifurcation parameter. Transcritical bifurcations are one example of zero-value eigenvalue bifurcations, whereby the dominant eigenvalue goes through zero as the system is forced through a critical transition. CSD was first described for fold bifurcations, which are another family of bifurcations with this property and dominate the literature. There are a few exceptions in infectious disease models where a Hopf bifurcation can be induced instead [46,47], although, in general, more complex infectious disease models can also be reduced to a transcritical bifurcation [48]. Fortunately, the trend in EWSs prior to fold, transcritical and Hopf bifurcations is similar in most cases [49], and so we can expect similar properties in EWSs for different classes of infectious disease models.

**Figure 1.**
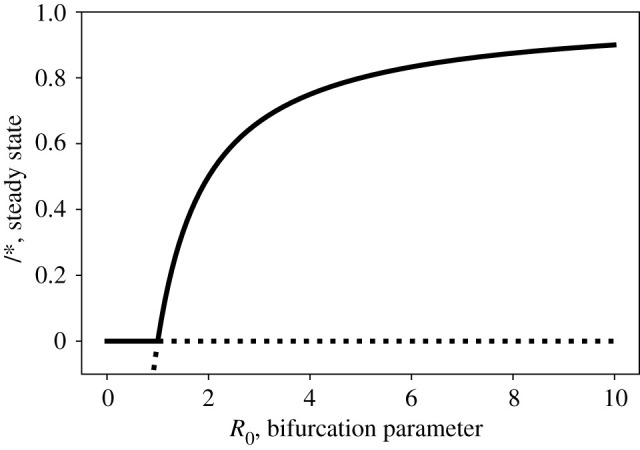
Bifurcation diagram of a typical epidemiological model. Transcritical zero-eigenvalue bifurcation diagram for an SIS model: bifurcation occurs at *R*_0_ = 1; if *R*_0_ < 1 then the disease-free state (I* = 0) is stable while if *R*_0_ > 1 then the endemic steady state (I* > 0) is stable.

Figure 1 demonstrates how the steady state of the system changes with the bifurcation parameter *R*_0_. To derive statistical indicators of disease transitions, the system can be perturbed analytically by gradually forcing parameters such as the transmission rate or vaccination rate over time, until the disease is either unsustainable (*R*_0_ < 1, disease elimination) or disease cases surge (*R*_0_ > 1, disease emergence). Changing parameters analytically effectively mimics public health campaigns such as hand washing, social distancing and vaccination programmes. Many studies also include an external force of infection term, *ɛ*, to allow for environmental spillover, which is particularly important for understanding the dynamics of disease emergence [21,23].

Under the stochastic formulation, the probability *P*(*I*, *t*) of having a prevalence of *I* at time *t* is given by the master equation,

2.2 $$\begin{array}{r} \frac{dP(I,t)}{dt}=(E_{I}^{-1}-1)T(I+1|I)P(I,t)+(E_{I}-1)T(I-1|I)P(I,t), \end{array}$$

where *T*(*I* + 1|*I*) = *β*((*N* − *I*)*I*/*N*) + *ɛ*(*N* − *I*) are the transition rates into the infected class and *T*(*I* − 1|*I*) = *γI* are the transition rates away from the infected class. The step operator $E_{I}^{k}$ is defined as $E_{I}^{k}f\;(I)=f\;(I+k)$, where *f* is an arbitrary function [50].

The theory of CSD relates to the fluctuations about a steady state, where the statistical properties of these fluctuations change on the approach to a critical transition. The linear noise approximation [50] has been applied to epidemiological systems to separate the fluctuations from the steady state [21], and assumes that the fluctuations, *ζ*, about the prevalence steady state, *ϕ* = I*/*N*, are expected to be of the order of *N*^−1/2^, agreeing with the central limit theorem

2.3 $$\frac{I}{N}=\phi (t)+\frac{\zeta }{\sqrt{N}}.$$

This linearization is only suitable when the number of infectious individuals is sufficiently large. For emerging diseases, where there are few infected individuals initially, the stochastic dynamics are highly non-Gaussian [23]. The birth–death–immigration process (BDI) has been adapted to the study of emerging diseases with births representing new infections, deaths representing the recovery of individuals and immigration the spillover of a pathogen from an external source; under this framework the transitions are *T*(*I* + 1|*I*) = *βI* + *ɛN* and *T*(*I* − 1|*I*) = *γI* [23]. The BDI process provides analytical intuition to behaviour of prevalence for emerging diseases, but it is limited in its applicability for established diseases at endemic steady state. The derivations of EWSs using the master equation follows similarly for both the BDI process and SIS model.

The Fokker–Planck equation describing the probability of observing fluctuation *ζ* at time *t* for the SIS model can be derived as

2.4 $$\begin{array}{rl} \frac{\partial \Pi (\zeta ,t)}{\partial t} & =(E_{I}^{-1}-1)T(I+1|I)P(I,t)\\ & \;+(E_{I}-1)T(I-1|I)P(I,t)-N^{1/2}\frac{d\phi }{dt}\frac{\partial \Pi (\zeta ,t)}{\partial \zeta }\;\\ & =-(\beta (1-2\phi )-\gamma )\frac{\partial \zeta \Pi }{\partial \zeta }+\frac{1}{2}(\beta (1-\phi )\phi +\gamma \phi )\frac{\partial^{2}\Pi }{\partial \zeta^{2}}, \end{array}$$

or equivalently in terms of the corresponding stochastic differential equation for *ζ*

$$d\zeta =(\beta (1-2\phi )-\gamma )\zeta dt+\sqrt{\beta \phi (1-\phi )+\gamma \phi }dW_{t}.$$

**Figure 2.**
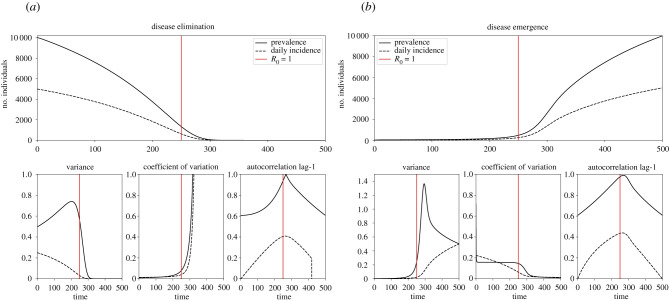
Time-series trends (theoretical). For the SIS model with external forcing the system is forced from the disease endemic state to the disease-free state ((*a*) disease elimination); and in reverse from the disease-free state ((*b*) disease emergence). The vertical red line denotes where the bifurcation occurs, when *t* = 250, *R*_0_ = 1. In both cases, the system is forced by slowly changing the transmission rate *β*(*t*) ∈ [0, 1] and keeping the recovery rate *γ* = 0.5 fixed, causing *R*_0_ to decrease from *R*_0_ = 2 to *R*_0_ = 0 (*a*) and in reverse *R*_0_ = 0 increases to *R*_0_ = 2 (*b*). The change in *R*_0_ happens at rate *p* = 1/500. Results for prevalence are shown by the solid black lines, and incidence by the dashed black lines. (*a*) Disease elimination theory and (*b*) disease emergence theory.

Analytically, statistical indicators can be deduced by taking moments of equation (2.4), such as evaluating the behaviour of variance of the fluctuations (illustrated in figure 2) to be

2.5 $$\begin{array}{rl} \frac{\partial {\left\langle \zeta^{2}\right\rangle }_{t}}{\partial t} & =\int_{-\infty }^{\infty }\zeta^{2}\frac{\partial \Pi }{\partial t}d\zeta \;\\ & =(\beta -\gamma -2\beta \phi )N\sigma^{2}+\beta (1-\phi )\phi +\gamma \phi , \end{array}$$

which can be simplified by evaluating at steady state, to obtain *var*(*ζ*) = (1/*N*)(1/*R*_0_) for the SIS model and var(*ζ*) = (*ɛ*/*γ*)/(1 − *R*_0_)^2^ for the BDI model. The steady-state assumption masks an important difficulty in the analysis of statistical indicators of critical transitions, namely that a system undergoing a critical transition is not at steady state. Nevertheless, this assumption can be viewed as a time-scale separation, whereby the bifurcation parameter varies much slower than the time it takes the system to equilibrate, and so, in principle, fast components can be eliminated [51,52]. While the time-scale separation argument provides some intuition about general trends, such a strict separation is not observed in epidemiological systems.

The coefficient of variation represents the ratio of the standard deviation to the mean, and therefore statistical properties of this EWS on the approach to a critical transition depend on whether the system is approaching: (i) disease elimination, where the mean infections are reducing, resulting in an asymptotically rising coefficient of variation (figure 2*a*), or (ii) disease emergence, where the mean infections are increasing (figure 2*b*). A consequence of using the linear noise approximation is that the mean of the fluctuations *ζ* is zero; for this reason derivations of the coefficient of variation can be made from prevalence $\sqrt{\langle I^{2}\rangle -{\left\langle I\right\rangle }^{2}}/\langle I\rangle$ by taking moments of *I* instead.

Another popular EWS of disease transitions is the lag-*τ* autocorrelation, which is predicted to rise prior to a critical transition from CSD. Derivations of auto-correlation have been made in the literature using the power spectrum and the Wiener–Khinchin theorem [21], and have been achieved using the Fokker–Planck equation. In particular, the lag-1 autocorrelation for prevalence is given by $\mathrm{ACF}=e^{-|\gamma (R_{0}(1-2\phi )-1)|}$ for the SIS model (shown in figure 2) and $\mathrm{ACF}=e^{(R_{0}-1)\gamma }$ for the BDI process at steady state.

Theoretical work on EWSs of disease transitions has been extended from this simple one-dimensional SIS model to a variety of epidemiological models, including the SIR (susceptible--infected--recovered) model [21,26], vector transmission models [22], models with vaccination dynamics [21,29,31,37], spatial metapopulation models [27], age-structured models [28] and higher dimensional models, including network-based [28,36,41], agent-based simulations [28] and coupled disease–behaviour dynamics on multiplex networks [29,37]. In these more complex systems, statistical properties of the fluctuations about prevalence have been investigated by extending the derivations described in this section.

Consistently the most popular EWSs studied, variance and lag-1 autocorrelation, are calculated on the fluctuations of prevalence-type data and rise prior to disease transitions in all models [21–23,27,31], shown in figure 2. While the coefficient of variation rises prior to disease elimination [21], it is flat and unchanging prior to disease emergence [23]. In contrast, the coefficient of variation, variance and lag-1 autocorrelation are constant and unchanging over time for a disease at the endemic steady state (see electronic supplementary material, figure S1). The non-monotonic trend in time-series statistics prior to reaching the disease transition can be used as the EWS of an upcoming bifurcation. The trend in some EWSs has been found to be sensitive to the source of noise driving the perturbation, where some statistics have been shown to decrease depending on the type of stochasticity present [24]. Notably, O’Regan & Burton [24] conclude that it is reasonable to assume that the variance and coefficient of variation will increase prior to disease elimination when the noise is external and affects a system as a whole.

The focus to date has been on determining whether the characteristics of CSD are exhibited in epidemiological transitions and more widely this work has shown evidence of CSD in transcritical bifurcation systems. A drawback present in these analytical studies is due to the type of epidemiological data collected, as prevalence-type data (*I*) are rarely collected except for closed systems (e.g. within a hospital setting) or during prevalence surveys (a sample of the population). Typically, incidence data are collected, which describe the number of new infections at each time; for example, number of new infections diagnosed each week or month. These data are also not without limitations, as many ‘new infections’ are not reported on the first day of infection, but after an individual has presented themselves at a healthcare facility. Additionally, many diseases are not notifiable (a disease that is required by law to be reported to government authorities), resulting in an inaccurate representation of disease.

Significant analytical progress has begun to understand whether EWSs calculated on incidence-type data are exhibited prior to a critical transition, and whether there are discrepancies between signals in prevalence-type data and those in incidence-type data. Under the BDI framework, a probability distribution for the *deaths* or recovery events can be derived analytically to understand the fluctuations about incidence. A common assumption is to monitor recoveries, assuming that cases are more likely to be reported towards the end of the infectious period. O’Dea & Drake [25] extend the BDI model in the context of emerging diseases to describe the distribution of new cases by adding an observation model into the framework. This can be constructed using the master equation (equation (2.2)), where *I* is the prevalence and *n*_*c*_ (new cases) is the total number of infectious individuals who are removed over an interval of length *t*,

2.6 $$\begin{array}{rl} \frac{dP(I,n_{c},t)}{dt} & =(E_{I}^{-1}-1)T(I+1,n_{c}|I,n_{c})P(I,n_{c},t)\\ & \;+(E_{I}E_{n_{c}}^{-1}-)T(I-1,n_{c}+1|I,n_{c})P(I,n_{c},t), \end{array}$$

where the transition rates are the same as the earlier BDI model (*T*(*I* + 1, *n*_*c*_|*I*, *n*_*c*_) = *T*(*I* + 1|*I*) and *T*(*I* − 1, *n*_*c*_ + 1|*I*, *n*_*c*_) = *T*(*I* − 1|*I*)) and the master equation contains step operators in terms of *I* (as previously) and *n*_*c*_. Including a monitoring scheme, by counting removal events, increases the dimension of the Markov process by 1, where new cases *n*_*c*_ increase by 1 each time there is a removal event. This can be solved by transforming the master equation into a multivariate generating function in terms of prevalence and new cases [53], and deriving analytical solutions of EWSs from the moments [44].

Other analytical work by Southall *et al.* [31] considers EWSs of disease elimination when calculated on incidence data, and compares their approach with previous studies and with the known traits of CSD when statistics are calculated on prevalence-type data. By considering stochastic models of infectious diseases, it is known that the new infection events (e.g. transmission events) can be estimated by a Poisson process with rates given by the transition probability *T*(*I* + 1|*I*). This generalized theory, which holds for many counting processes outside the scope of epidemiology, can be used to find the EWSs from the statistical signatures of a Poisson distributed variable. Namely, if new cases happen at a rate approximately equal to *λ*(*t*) = *T*(*I* + 1|*I*)Δ*t*, then the variance and mean are equal and given by *λ*(*t*). This result says that when the mean is increasing, such as prior to disease emergence, then the variance in incidence will increase before the critical transition, as expected by CSD and supporting previous work [25]. However, when the mean is decreasing, such as prior to disease elimination, then the variance of incidence will decrease before the critical transition. This result signifies that the rising property of variance prior to a disease transition, which was found for prevalence-type data, is not always exhibited prior to disease elimination and depends on the data collected (illustrated in figure 2*a*).

Furthermore, a lower lag-1 autocorrelation is observed in incidence data compared with prevalence data (figure 2). A formal analysis of the reduced autocorrelation of incidence data relative to prevalence data is beyond the scope of this review. However, it can be heuristically seen by inspecting the transition rates of the BDI model for *n*_*c*_ and *I*, which only explicitly depend on *I* (equation (2.6)). Therefore, the dependence of new incidence on previous incidence is indirect (via the prevalence *I*, which depends on the entire history of infections and recoveries). For prevalence, the dependence is direct, suggesting prevalence data are more correlated than incidence data.

## 3. Computing EWSs from disease data

The calculation of EWSs from real-world data requires suitable pre-processing, such as the *detrending* of the data to remove the mean (steady state) and obtain the fluctuations. In simulation studies, this process can be done by removing the average over replicate realizations, and it has been shown that stochastic simulations produced using the Gillespie algorithm match the theoretical predictions of EWSs shown in figure 2 [25,27,31,35]. In practice, without the availability of true replicates, *Gaussian detrending* is often implemented. Gaussian detrending is a moving average technique, which removes a weighted mean over a selected window size, where the weights are taken from a Gaussian kernel with a user-inputted standard deviation. This method not only requires the user to select a suitable choice of window size and standard deviation, but also makes the assumption that the data are ergodic. This raises a key challenge with this technique, as ergodicity only holds for stationary time series; however, these methods will be implemented on data that are believed to be approaching a critical transition. For this reason, the choice of the window size and the speed a disease is approaching a critical transition are interlinked when deciding if the assumptions of Gaussian detrending are appropriate. In particular, O’Regan & Drake [21] discussed the limitations of Gaussian detrending for diseases that decline rapidly, finding that, even for slowly changing diseases, smaller window sizes did not capture the fluctuations and larger window sizes did not successfully remove the slowly varying trend. Even with its recognized limitations, Gaussian detrending is a popular method in the EWS literature for disease transitions [21,22,33,40].

Recent work has begun to develop a specific detrending method for epidemiological data. Dessavre *et al.* [27] present a spatial detrending approach, which removes the mean over multiple populations to obtain the fluctuations. Unlike Gaussian detrending, this approach does not require any hyperparamaters to be inputted; however, it does assume spatial ergodicity, i.e. all subpopulations are similar. The use of spatial data to overcome some limitations in detrending is promising, particularly for fine-scale spatial data, such as within a county or state where the spatial ergodicity assumption is suitable. Furthermore, O’Dea & Drake [25] found that statistics calculated over multiple heterogeneous realisations, where the parameter set for each realization was sampled randomly, corresponded well with the analytical results. This simulated study could be thought of as calculating EWSs between many non-identical locations, supporting evidence of spatial detrending.

In addition, there is the added challenge of removing periodic trends in the time series to obtain the residuals. Many infectious diseases exhibit seasonal forcing due to climate and human behaviour, which may dominate the behaviour of EWSs close to the critical transition. However, Miller *et al.* [33] found that it was not necessary to seasonally detrend the time series first, noting that autocorrelation performed worse with seasonal decomposition than Gaussian detrending. It was shown that variance was insensitive to the type of detrending (Gaussian, seasonally decomposition or differencing), although the performance worsened with all detrending methods when the data had high levels of periodic forcing.

## 4. Performance of EWSs

Simulation studies can provide an understanding of the performance of each EWS for different transitions. Error rates of EWSs can be visualized by receiver operating characteristic (ROC) curves of each signal. The sensitivity, or true-positive rate, can be measured as the proportion of simulations going through a transition which are correctly identified. The specificity, on the other hand, or true-negative rate, gives the proportion of simulations not going through a transition that are correctly identified. The latter group of simulations are often referred to as the null model, and EWSs calculated on these data should not signal a critical transition. For disease emergence, a high sensitivity is of critical importance to provide a high confidence of identifying all disease emergence risks. By contrast, for disease elimination, a high specificity is required to minimize falsely detecting disease elimination in cases where it is not present.

Many methods have been proposed to measure the performance of EWSs, such as thresholding EWSs with a constant value [27,28], thresholding with the long-run standard deviation [13] or using Kendall’s *τ* statistic. Kendall’s *τ* statistic is the most popular approach in the literature [21,22,31,33–35,40], as it gives a quantitative measure of the increasing or decreasing trend of the EWS. However, the use of Kendall’s *τ* score can be problematic as it describes the overall trend over the time period considered, with the challenge being selecting an appropriate time period. Even if the EWS rapidly increases and then decreases slightly, the score may return a value of zero (indicating a constant trend) or even a negative score (decreasing trend). Simulation studies have considered how the performance of an EWS behaves over different time intervals [27,28], with one study finding that the variance and autocorrelation lag-1 did not become predictive until 2 years before the estimated transition [28], further indicating that the choice of time interval used to calculate Kendall’s *τ* statistic can impact results.

There exist various ways to determine the best time period for empirical studies on historical data, such as using an expert’s opinion to infer the time of transition [38], using an appropriate mathematical model to retrospectively identify when the effective reproduction number is 1 [28] or approximating when the system underwent a critical transition using the rate of change of incidence [40]. In these examples, Kendall’s *τ* score can be calculated on a reasonable time period before the estimated transition. A key unanswered question is how much data should be used to calculate Kendall’s *τ* score for a real-time analysis, where the true critical transition is unknown and not yet reached.

The choice of null model assesses the strength of an EWS under different conditions, and many studies choose a model at steady state where the value of *R*_0_ is fixed over time [21,22,28,31,33,35]. A steady-state null model will describe how good an EWS is at identifying the difference between a system undergoing a bifurcation versus a system at steady state. A tougher test is measuring an EWS's ability to identify when the system is approaching a critical transition versus when the system is changing but not bifurcating. EWSs which perform very well when comparing disease elimination or emergence to a steady-state null model can struggle to distinguish between the disease transition and a time series which was changing but not bifurcating [27,39]. This highlights a serious limitation for detecting disease transitions when using EWSs in practice. Ideally, EWSs need to be capable of distinguishing between increased transmission and increased reporting, given the latter type of data may not be bifurcating. The choice of an appropriate null model can change the perceived view of accurate EWSs and the trade-off between sensitivity and specificity will depend on whether distinguishing disease transitions and steady state is enough.

### 4.1. How *early* can EWSs identify the disease transition?

Research addressing the first time when the trend of an EWS is significant, which we name *time of detection*, is limited. The time of detection refers to detecting the statistical signatures of CSD, rather than the critical transition itself. Reporting the time when there is evidence of an upcoming bifurcation will allow policy makers to intervene and change the current direction. For studies where the bifurcation point is known, the time of detection can be used to find the *lead time* of each EWS. The more advanced the warning of an approaching transition (e.g. the more lead time), the more valuable an EWS will be in practice.

The logistic composite measure from Brett & Rohani [39] is an EWS-based approach which offers the time of detection. This method is designed for real-time implementation, where the logistic measure is updated as new data are observed, and a detection is triggered when the threshold criteria are exceeded. This method not only attempts to distinguish between the null model and disease emergence but also offers the first time there is significant evidence of an approaching critical transition. Their technique is tested on empirical studies of pertussis, mumps, dengue and plague. Using synthetic data, they found that the composite measure outperformed a single EWS, and could inform disease emergence of pertussis in 100% of states which did experience re-emergence, and falsely detected 30–50% states as disease re-emergence. The presence of sporadic outbreaks might explain this mild specificity score; however, presenting this weakness is highly valuable information for decision-making. This study focused exclusively on anticipating disease emergence. Given the observed theoretical differences preceding disease elimination (see table 1 and figure 2), it is extremely unlikely that the fitted weights would also be appropriate for elimination. Constructing a composite measure to serve as a leading indicator of disease elimination thus remains an open research topic.

Drake & Griffen [13] proposed that an EWS is significant if it exceeds two long-run standard deviations, and the time of detection is given by the first time the signal exceeds this threshold. This method can be computed on a composition of EWSs, after a normalization process, and the threshold criterion (referred to as 2*σ*) is updated as more data become available by calculating the long-run standard deviation of the composite measure. This general dynamic threshold is a key advantage of this method when compared with the constant threshold used in the logistic composite measure. However, although the 2*σ* method has been demonstrated to detect the onset of environmental deterioration [13], population collapse [54], recovery of an ecosystem [55] and the emergence of COVID-19 [43], it has not been formally validated with simulation studies of infectious data, which remains an open topic.

Other work in identifying the time of detection using EWSs has not been formally validated with simulation studies, so the sensitivity and specificity of these approaches are unknown. Without further investigation, it is not known if these methodologies will work in general for other diseases or with different types of collected data. One method fitted a hyperbolic equation to the time evolution of the coefficient of variation to identify when the statistic diverges, as this is recognized as the location of the threshold analytically [32]. When tested on data going through a bifurcation, on average the time of detection occurred before the true bifurcation point, and under-reporting of case data under a binomial framework has little effect on the results.

Another method that uses bootstrapped samples to estimate the statistical significance of an observed Kendall’s *τ* statistic has proven popular in the literature [10,15,56–59] but also controversial [15,56,59]. An empirical study tested if EWSs could indicate malaria resurgence in a historical dataset from Kericho, Kenya [38]; using bootstrapping with Kendall’s *τ* score over increasing amounts of data to calculate the *p*-value of the score over time. Evidence of CSD was said to be detected when the *p*-value became significant at 0.05. Under this framework, the resurgence of malaria was predicted with a 24-month lead time with autocorrelation lag-1 and a six-month lead time with variance. However, this method has not been tested on time-series data which are not going through a critical transition, i.e. the method’s specificity remains unknown.

Other studies use the Brock–Dechert–Scheinkman (BDS) test [60], which detects nonlinear serial dependence in time series. Dakos *et al.* [59] reviewed the use of the BDS test in the context of EWSs with bootstrapping and *p*-values, highlighting the issue of approximating the null distribution with this technique. Many articles have warned about reordering the time series to create the null distribution, while maintaining the variance and mean of the observed time series, as it impacts the natural autocorrelation in the time series [56]. Some have instead suggested using a model-based approach to generate a null distribution with the same variance, mean and autocorrelation using an autoregressive model [15]. However, White *et al.* [61] questioned estimating the null distribution for simulation-based models, saying that this is an inappropriate use of statistical significance tests and concluding that *p*-values should not be used to interpret results.

### 4.2. How do EWSs behave in a data-poor setting?

Analytical studies have shown that CSD behaviour is present in disease models prior to a transition, but the exact behaviour depends on the transition. Calculations of EWSs on real-world data, in contrast to simulation studies, are subject to reporting errors, and are relatively short length, owing to the frequency of observations.

Simulation studies have tested the robustness of EWSs to reporting errors, often determining the change in the AUC (area under the ROC curve) metric when taking binomial samples from the data. For disease elimination, the coefficient of variation was found to be the most robust EWS under the effects of observation error, while the variance and autocorrelation were shown to be sensitive to under-reporting [21,32]. Data which are subjected to reporting errors through negative binomial sampling can be used to investigate the effects of reporting probabilities where the number of new cases can exceed the true number, representing over-reporting or false-positive diagnoses of cases, as well as incorporating some dispersion and clustering in case ascertainment. Indicators of disease emergence were found to be sensitive to overdispersion, particularly when the reporting error is highly overdispersed and, in contrast to studies on disease elimination, variance was the most robust and coefficient of variation the most impacted by reporting [35]. Furthermore, O’Dea & Drake [25] defined a robust indicator to be sensitive to the difference between the transmission and recovery rates of the system and insensitive to changing reporting probabilities, finding that variance and coefficient of variation were poor EWSs under the first criterion and autocorrelation lag-1 poor owing to the latter criteria.

To date, the performance of EWSs has not been investigated when there are systematic biases in the sampling processes; for example, owing to age-specific disease severity, or increased under-reporting in specific demographic groupings. Understanding the impact of biased sampling on EWSs is an essential avenue for future research.

Furthermore, insufficient data resolution is often prevalent in epidemiological data collection; for example, the World Health Organization reports yearly cases of neglected tropical diseases which are targeted for elimination by 2030 [62]. It is unclear if EWSs can be used to assess the progress towards these goals with such short time series (for example, case data of human African trypanosomiasis are reported yearly and are only available since 2000 at a high spatial resolution). Although synthetic studies have shown the suitability of some EWSs in large evenly spaced datasets [21,27,31,35], lower bounds on the necessary data frequency remain an open question. Creating evenly spaced data from irregularly sampled data may introduce artificial autocorrelation [56], although studies have shown that aggregation of case reports into weekly, bi-weekly and four-weekly did not impact the performance of EWSs [35]. In a study on seasonal outbreaks of bubonic plague in Madagascar, the outbreak was detected 27 days after the first reported case, giving a 30-day lead time before the major outbreak in late September [39]. This study used high-resolution daily case counts and the result suggests that with sufficient data resolution CSD may be detectable over comparably short time scales. Investigating the quality of data required, for example whether the temporal range of a dataset is more important than the temporal resolution or sampling frequency (e.g. 100 months of monthly data versus 100 weeks of weekly data), should be the subject of further work.

Although longitudinal data can be noisy and low resolution, other high-dimensional information, such as the health zone a case was reported in, can be available. Dynamical spatial data can be represented using network models, and spatial EWSs can be investigated on the network structure which offers the potential for regional disease transitions to be identified [27,28,36,41].

Additionally, there is limited work investigating the assumption that CSD is only exhibited in data that are slowly changing [12,63]. Emerging diseases can surge rapidly on a fast time scale, for instance the emergence of the SARS-COV-2 pathogen which causes the disease COVID-19. Although COVID-19 has been the focus of recent studies on EWSs [40–43], further work is required to understand the performance of EWSs subject to factors affecting the speed of a disease, sampling frequency and time scale of data. Indeed, the speed of disease has been explored in simulation studies, finding that the rising autocorrelation trend typically reaches 1 at the transition unless the system has a fast speed of emergence [28].

Twitter data can be available at a high frequency and spatial resolution, and these attributes offer the potential to overcome some of the limitations when using incidence- or prevalence-type data. Research by Pananos *et al.* [34] geocoded Twitter streams monitoring tweets about the measles–mumps–rubella vaccine, and used EWSs to detect the rise in vaccine hesitancy. The analysis on vaccine sentiment was found to exhibit traits of CSD prior to the Disneyland, California measles outbreak, providing evidence that behavioural dynamics can also help inform disease transitions and have the potential to expand the current toolbox of EWSs.

## 5. Case study: emergence of pertussis in the USA (1992–2007)

Here, we present a demonstration of how EWSs behaved for pertussis in the USA between 1992 and 2007. Pertussis is a highly contagious infectious disease, which became preventable by vaccine in 1942, and has been a notifiable disease in the USA since 1922 [64]. The high vaccine uptake for pertussis resulted in a 99% reduction in cases between 1934 and 1976 [65]; however, during the 1980s incidence began rising rapidly [66]. Today, pertussis is considered one of the most poorly controlled vaccine-preventable diseases in the USA [67], and cases in 2012 were the highest recorded since 1955 [65]. Although the USA has a high vaccine uptake (nationally increased from 64.4% to 95.9% between 1979 and 1999 [68]), communities with higher vaccine exemption rates recorded higher pertussis rates and between 59% and 93% of cases occurred in the unvaccinated population during eight of the outbreaks [69], with large outbreaks occurring in 2004, 2010, 2012 and 2014 [67].

We are interested in whether the pertussis outbreaks in the early twenty-first century can be identified using EWSs. We analyse the results from EWSs for different US states, where states were categorized into very-high-risk, high-risk, medium-risk or low-risk burden of incidence, by calculating the 90th, 50–90th, 10–50th, below 10th percentiles, respectively, from the observed yearly incidence records. The states with the highest number of years in the very-high-risk category between 1992 and 2007 were Vermont (14 years), Massachusetts (10 years), New Hampshire (9 years) and Idaho (9 years), and the states most often in the low-risk category were Mississippi (12 years), Louisiana (12 years), West Virginia (7 years) and Georgia (7 years). This dataset has previously been analysed, where states were classified as either emerging or not using a one-sided *t*-test [39]. In particular, the geographical variations of pertussis burden from both methodologies are in agreement for very-high-risk and low-risk states.

**Figure 3.**
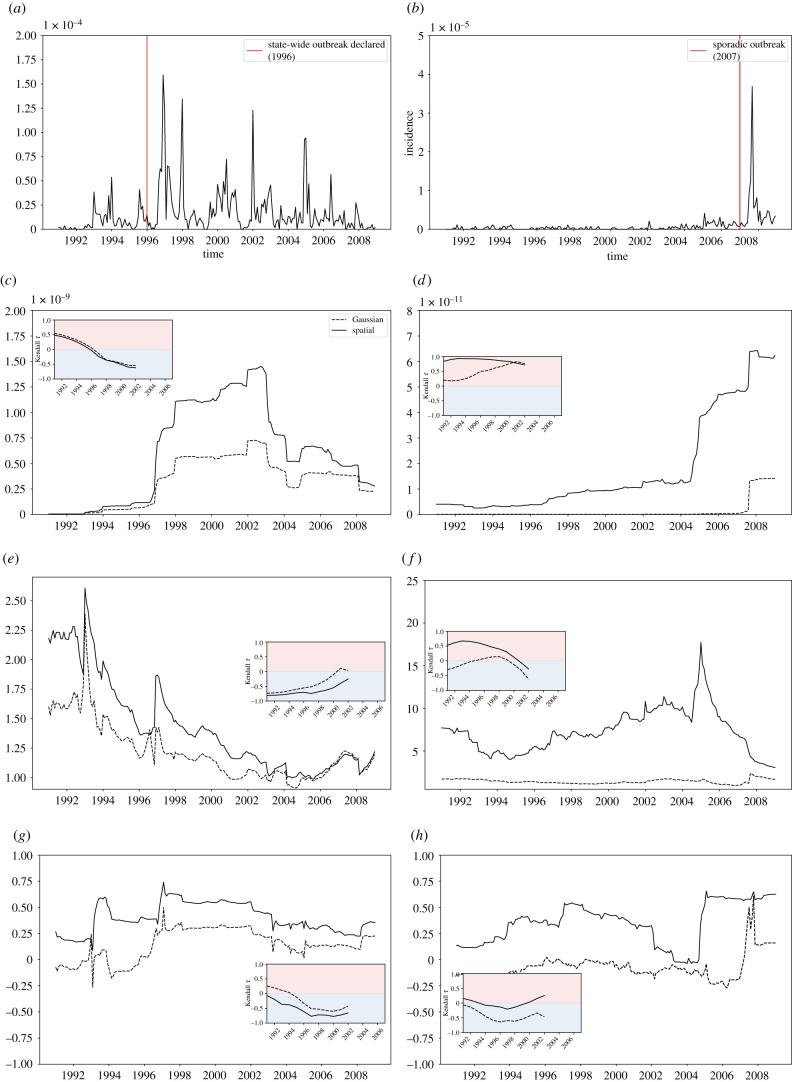
Time-series trends of EWSs. EWSs calculated on monthly pertussis incidence data. (*a,c,e,g*) show trends for Vermont (highest incidence burden between 1992 and 2007). (*b,d,f,h*) show trends for Mississippi (lowest incidence burden between 1992 and 2007). EWSs calculated using spatial detrending are shown by solid lines, and with Gaussian detrending by dashed lines (window size 10% of time-series length (window size = 70), standard deviation of Gaussian filter calculated using Silverman’s rule of thumb (s.d. = 4.55, mean over states)). EWSs were calculated on detrended time series using moving averages (right window) with window size 10% of time series, data prior to 1991 was cut off after calculations. Inset shows Kendall’s *τ* score for each EWS on varying amounts of data up to 2007. Kendall’s *τ* at time point *t* is calculated over the window EWS ∈ [*t*, 2007]. (*a*) Incidence (Vermont). (*b*) Incidence (Mississippi). (*c*) Variance (Vermont). (*d*) Variance (Mississippi). (*e*) Coefficient of variation (Vermont). (*f*) Coefficient of variation (Mississippi). (*g*) Autocorrelation lag-1 (Vermont). (*h*) Autocorrelation lag-1 (Mississippi).

To address how EWSs behave in data-poor settings, where cases may be reported less frequently, we focus our analysis on the state with the highest burden of incidence (Vermont, figure 3, left column) and the state with the lowest burden of incidence (Mississippi, figure 3*a,c,e,g*). Mississippi has the highest childhood vaccination rate in the USA [70] and is one of two US states (between 1992 and 2007) which do not allow non-medical vaccine exemption. However, incidence of pertussis has increased recently with sporadic outbreaks occurring in 2007 and cases rising to a peak in 2014 [71]. In 1996, Vermont reported a state-wide outbreak of pertussis [72], and another epidemic was declared in 2012.

**Figure 4.**
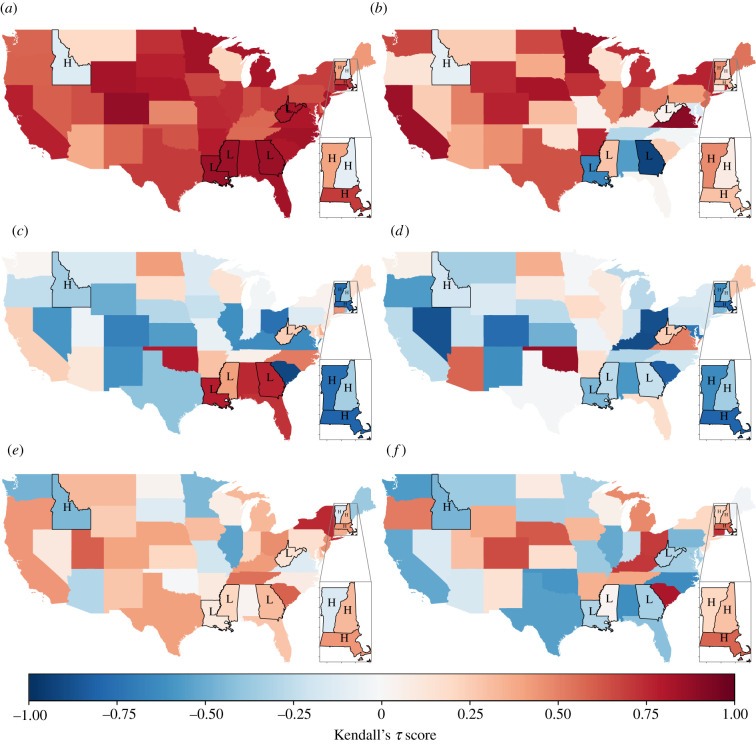
Kendall’s *τ* score for variance, coefficient of variation (CoV) and autocorrelation lag-1 (AC(1)). Kendall’s *τ* score calculated on EWSs for each state between 1992 and 2007. A score near 1 indicates an increasing trend in the EWS, while a score near −1 indicates a decreasing trend. (*a,c,e*) show EWSs calculated on spatial-detrending data; (*b,d,f*) show EWSs calculated on Gaussian-detrending data as described in electronic supplementary material, figure S2. States labelled ‘H’ indicate the four states of highest incidence (Vermont, Massachusetts, New Hampshire, Idaho); states labelled ‘L’ indicate the four states with lowest incidence during this period (Mississippi, Louisiana, West Virginia, Georgia). (*a*) Variance (spatial detrending). (*b*) Variance (Gaussian detrending). (*c*) CoV (spatial detrending). (*d*) CoV (Gaussian detrending). (*e*) AC(1) (spatial detrending). (*f*) AC(1) (Gaussian detrending).

Figure 3 shows the time changing behaviour of the variance, coefficient of variation and autocorrelation lag-1 for Vermont and Mississippi (electronic supplementary material, figure S2 gives the trends for all eight states in the highest and lowest categories). The vertical red line corresponds to external information on the year of a state-wide outbreak, and is not used in our analysis of EWSs. Monthly recorded cases of pertussis were detrended using spatial detrending (solid lines) and Gaussian detrending (dashed lines), where the hyperparameters for Gaussian detrending were chosen with a window size of 10% of the time series and the standard deviation found using Silverman’s rule of thumb [73]. To calculate the statistics, a moving average technique was implemented on the detrended data, with a window size of 25% of the time series. The inset shows Kendall’s *τ* score calculated on each EWS over the time frame $\left[\bar{t},2007\right]$ for $\bar{t}\in [1991,2003]$, and figure 4 shows Kendall’s *τ* score when calculated over the entire time period (i.e. when $\bar{t}=1991$) for each state and EWS.

Figure 3*c*,*e*,*g* shows that the EWSs and Kendall’s *τ* results were largely consistent across detrending types for regions of a very high burden of incidence (also shown in figure 4; states labelled ‘H’), while in regions of low incidence (figure 3*d*,*f* ,*h* and figure 4 labelled ‘L’) the results are contrasting between spatial and Gaussian detrending. Notably, the time evolution of variance in Mississippi (figure 3*d*) visually increases for Gaussian and spatial detrending; for spatial detrending this corresponds with a Kendall’s *τ* score ≥0.75, signifying the increasing trend, but for Gaussian detrending over the same time period the score ≈0.25 (e.g. constant trend). The overall agreement between spatial and Guassian detrending for each state is shown in electronic supplementary material, figure S5. In particular, we find that variance is the most sensitive to detrending type, and autocorrelation lag-1 the least. Spatial detrending was conducted over all states in continental USA (i.e. excluding Alaska and Hawaii), although we stress that, owing to the geographical unevenness of pertussis, this does not satisfy the spatial ergodicity condition. Further work is needed to test a selective-spatial detrending approach, where US states of similar pertussis or vaccination rates are detrended together.

The inset figures (figure 3) investigate the sensitivity of Kendall’s *τ* score with respect to the time frame used. In figure 3*c*, Kendall’s *τ* score changes from weakly increasing over the whole time series of variance to weakly decreasing when just including the previous 4 years, reflecting the increasing behaviour of variance from 1995 to 2003, before it falls. This could lead to misleading conclusions if the interval to calculate Kendall’s *τ* score is not chosen carefully. Further studies are needed to understand the sensitivity of Kendall’s *τ* score, addressing the performance of EWSs for different detrending types and different lengths of time-series data available.

To provide a quantitative analysis of how Kendall’s *τ* score changes when subject to less frequent reporting, we aggregate the monthly reported data (length 203) into: bimonthly (every two months, length 101); quarterly (every six months, length 67); triannually (every four months, length 50); biannually (every six months, length 33); yearly (every 12 months, length 16); biennially (every 2 years, length 8); triennially (every 3 years, length 6) and quinquennially (every 5 years, length 3). For each choice of data aggregation, we calculate the EWS with moving average techniques, using window sizes ranging from 5% to 75% of the length of the aggregated time series.

For the high-risk burden state, Vermont, Kendall’s *τ* score is insensitive to data aggregation for variance and coefficient of variation, and insensitive to the detrending method for all three statistics considered (electronic supplementary material, figure S3). However, the window size of the moving average impacts the Kendall’s *τ* score, so that for window sizes between 15% and 55% of the time-series length the variance increases (Kendall’s *τ* score near 1), while for window sizes greater than 55% variance strongly decreases (Kendall’s *τ* score near −1). The choice of window size has been investigated by Lenton *et al.* [14] as well as by Kaur *et al*. [40] in their study on EWSs of COVID-19 emergence, finding that large window sizes altered the results. In summary for Vermont, variance is strongly increasing for most choices of window sizes and aggregation, coefficient of variation is weakly decreasing, and autocorrelation lag-1 has a mixed response which perhaps can be explained by the stochastic nature of pertussis in Vermont; the former two indicate characteristics of disease emergence from CSD.

By contrast, all EWSs are sensitive to detrending for Mississippi, and detrending can influence whether the observed trend is increasing or decreasing. However, variance and coefficient of variation are insensitive to time aggregation, and results are consistent when the window size is between 15% and 55%. No conclusions on the status of pertussis in Mississippi can be drawn from this analysis, with spatial detrending suggesting that all EWSs are increasing, perhaps indicating disease elimination (although we would expect variance to decrease prior to elimination [31]). However, results from Gaussian detrending—variance is weakly increasing or flat, coefficient of variation is weakly decreasing or flat and autocorrelation lag-1 is weakly increasing—would suggest that Mississippi is undergoing disease emergence.

## 6. Discussion

EWSs offer a real-time signal of an impending disease transition; however, to use EWSs reliably in a control-management framework, all limitations need to be identified and communicated clearly with public health officials. Progress has been made in numerous areas, from theoretically motivated studies identifying how EWSs behave for different disease dynamics, data collected or stochasticity present to numerical studies testing pre-processing techniques and understanding the limits of EWSs with imperfect data. There remain challenges and limitations to all methods reviewed in this paper. In particular, we identified a few key areas, such as drop in the specificity of EWSs when tested on data which are changing but not approaching a transition, and the unknown specificity of some of the detection methods presented. On the theoretical side, questions remain how best to quantify EWS uncertainty when using moving-window estimators, and how the speed of a disease and sampling frequency are interlinked when using a moving window.

In this review, we have highlighted key theoretical differences in EWS for emergence and elimination transitions but also the differences in objectives, requiring a high sensitivity for EWS of disease emergence compared with a high specificity for disease elimination. Furthermore, the trend of some EWSs changes for different data types. To minimize error rates and improve the performance of EWSs, there is the potential to apply EWSs to multiple datasets simultaneously, such as with prevalence surveys, reported case data and Twitter streams, to provide an overview of the status of disease. If all three data types are consistent with their detection of a disease transition, then that provides greater confidence in the result.

Determining which EWSs are best suited for different diseases when faced with a real system remains a challenging problem. From the EWSs reviewed here, variance is the most sensitive to reporting errors prior to disease elimination, coefficient of variation is the least robust indicator prior to disease emergence and lag-1 autocorrelation is sensitive to the frequency of data. Despite this, the declining trend of variance prior to disease elimination and the rising trend prior to disease emergence is a particularly valuable feature; monitoring the trend of variance could distinguish between disease transitions and would be beneficial for a disease which is close to elimination and at risk of resurgence. Other potential EWSs exist and have been the subject of theoretical studies; significant progress has begun to find the optimal combination of multiple EWSs to give a single measure. A composition could be adapted for different diseases and data types, and has the potential to achieve an increased indication of a disease transition compared with a single EWS.

The application of these methods for use by stakeholders or programme managers in decision-making is a key goal of this field. For disease emergence, early detection could provide sufficient time to allow control measures to be implemented before the tipping point is met. Even if there is no lead time of a bifurcation, i.e. an EWS becomes significant after the critical transition, there is still evidence of the disease transition which is clearly important for policy. If the threshold has already been crossed, EWSs can inform policy makers whether to maintain the current control and if the disease is on the path to elimination.

When considering the practicalities of using EWSs to inform disease management, it is necessary to understand the implications of bifurcation delay. Even once the disease elimination transition has been crossed, if control measures are maintained disease elimination becomes inevitable—it is not immediate. Many stakeholders and public health officials will want to know how long control needs to be maintained to achieve this goal [32]. Distinguishing the phase of the bifurcation anticipated by the EWS from the tail phase post-transition is crucial. In particular, during the tail phase of disease elimination, the control needs to maintained to ensure that *R*_0_ < 1 leads to zero infections. Alternatively, for disease emergence the bifurcation delay is the time between the critical transition and the start of an epidemic; often a series of minor outbreaks occur during this period which may also be detected by an EWS [74].

How EWS analysis can be incorporated into a decision-making framework remains an open question. The logistic composite method [39] is the first robust EWS-based method which offers the potential for real-time monitoring of incoming data streams of reported cases, which could trigger an alarm when the statistical signatures of CSD are identified. This method presents a possible system for using EWSs during surveillance of infectious diseases.

In this paper, we have sought to review EWSs, which have been proposed as a model-independent method for detecting disease elimination and disease emergence, before the disease transition is reached. The majority of the current literature has focused on understanding if EWSs can be used to detect disease transitions in incidence- or prevalence-type data. However, one key issue is addressing how EWSs behave in data-poor settings, and the importance of identifying all limitations of EWSs before they can be used reliably for decision-making. This review has shown that not all EWSs behave the same in every setting, and their performance at detecting CSD depends on the disease transition and on the type of data used. The generality of a EWS is its greatest strength; other than knowing the expected type of transition (elimination or emergence), EWSs make few assumptions about the underlying dynamics of the disease. However, before an effective toolbox of EWSs can provide reliable guidance for disease management some further research (through a combination of theoretical and empirical studies) is necessary to address the ongoing challenges identified in this review. How does the speed of the disease approaching the transition impact results? What time frame should be considered and what quantity/frequency of time-series data is required to be able to make reliable decisions? How should imperfections in the data be dealt with? The development of EWSs relies on tackling these weaknesses, and other unknown limitations which can only be discovered after further studies on real-world data.

## Data accessibility

Data and code available on https://github.com/ersouthall/disease_transitions.
